# Mind in Metabolism – A Comprehensive Literature Review on Diabetes and its Connections to Obsessive Compulsive Disorder, Schizophrenia, and Bipolar Disorder

**DOI:** 10.1007/s11892-024-01564-0

**Published:** 2024-12-09

**Authors:** Anja Cannon, Caitlon Jacoby, Allyson S. Hughes

**Affiliations:** Department of Primary Care, Diabetes Institute, Institute to Advance Health Equity (ADVANCE), Ohio University Heritage College of Osteopathic Medicine, 102 W Green Dr, Athens, OH 45701 USA

**Keywords:** Type 1 diabetes, Type 2 diabetes, Mental health, Bipolar disorder, Schizophrenia, Obsessive compulsive disorder

## Abstract

**Purpose of Review:**

The co-occurrence of diabetes and mental illnesses such as bipolar disorder (BD), obsessive-compulsive disorder (OCD), and schizophrenia creates significant barriers for both people with diabetes (PWD) and their healthcare teams. This literature review provides an analysis of the relationship between diabetes and mental illnesses through exploring epidemiology, shared risk factors, and clinical implications. The aim is to enhance the understanding of these complex comorbidities to guide and improve future research and clinical practice.

**Recent Findings:**

Recent research suggests a strong link between mental illness, metabolic syndrome, and diabetes. Studies show that BD has a robust relationship with metabolic disease and the antipsychotic medications used in treatment for many mental illnesses are strongly associated with weight gain and metabolic disease. However, there is limited research exploring the bidirectional relationship that diabetes has with BD, schizophrenia, and OCD.

**Summary:**

While research exists on the link between diabetes and mental conditions such as depression and anxiety, little research has examined schizophrenia, OCD and BD. The findings noted in this review suggest gaps in treatment options, healthcare services, and social support. While this paper provides a foundation for future progress, advancement in this field will require a collaborative effort from researchers, healthcare professionals, and community outreach programs to effectively close the gaps in care noted in these patient populations.

## Introduction

Although people with diabetes (PWD) are living longer due to medical developments and innovation, there remains a distinctive difference in the life expectancy of those living with comorbid mental illness. The prevalence of diabetes in 20–79 years of age globally is 10.5% and is projected to rise to 12.2% in 2045 [1]. In the U.S., projections suggest that the number of adults diagnosed with diabetes will nearly triple by 2060 at about 60.6 million people [2]. In addition, the American Diabetes Association (ADA) approximates that 1 in 4 people in the U.S. have undiagnosed diabetes [3, 4]. PWD are at a greater risk for developing mental illnesses [5]. A third of PWD have concomitant psychological and/or social complications that hinder the self-management of their diabetes [6]. Due to the prevalence of diabetes, it is important for healthcare professionals to understand how mental health impacts diabetes. A recent European survey found that 75% of PWD who felt they required support from a mental health specialist could not access it, and they expressed that available support lacked education on the emotional challenges of diabetes [7].

Diabetes psychosocial challenges are so impactful that the term diabetes distress was created to describe the negative emotional impacts the disease has on individuals [8] Diabetes distress factors include a sense of powerlessness and hopelessness, fear of complications, and exhaustion from the constant management tasks [9, 10]. Importantly, PWD are at a greater risk for developing mental illnesses. Those with a severe mental illness (such as schizophrenia) have a life expectancy that is 25 years shorter on average than the general population (typically from pathological illnesses and not suicide) [11]. Conversely, individuals with mental illness are susceptible to unhealthy lifestyle factors, like obesity, poor diet, and lack of exercise, that can lead to development or exacerbation of diabetes [5]. Many pharmacologic treatments for mental illnesses produce markedly higher rates of diabetes, especially antipsychotics and antidepressants, which are associated with weight gain and heightened insulin resistance which has been theorized to be the underlying cause of their association with diabetes [11]. Nevertheless, instances of newly developed diabetes without concurrent weight gain have also been documented, and the exact mechanism underlying this relationship is unclear [12]. As we understand more about the bidirectional relationship between diabetes and mental health outcomes, healthcare providers need to adopt a more holistic and mindful approach in their management of the disease [7, 11].

There are different classifications and categories of increased risk for diabetes. Type 1 diabetes (T1D) is an autoimmune disease that attacks the cells of the pancreas responsible for producing insulin. Type 2 diabetes (T2D) encompasses 90–95% of all diabetes and is a disease of peripheral insulin resistance usually accompanied by relative insulin deficiency [13]. Both diseases require continuous monitoring of blood sugar levels to avoid instances of hyper and hypoglycemic events. Prediabetes is a state of atypical carbohydrate metabolism like in T2D, but glucose levels are not high enough to qualify as T2D. According to the ADA, prediabetes should be considered a risk factor for the development of T2D [13]. Like prediabetes, another category that is considered a risk factor for developing T2D is metabolic syndrome which is defined as an aggregate of measured metabolic abnormalities including hypertension, dyslipidemia, central obesity, and impaired glucose metabolism [14]. Diabetes is associated with increased risk of numerous chronic health conditions such as cancers, cognitive impairment, nonalcoholic fatty liver disease, cardiovascular disease, and many others [13]. On top of this, diabetes can incur acute complications, like diabetic ketoacidosis (DKA), hyperglycemic hyperosmolar state (HHS), lactic acidosis (LA), and hypoglycemia. Although these are preventable states, they add markedly to total healthcare costs due to their high morbidity, hospitalizations, and mortality rates [15]. Thus, decreasing the prevalence of diabetes and optimizing self-management of the disease is crucial.

The relationship between diabetes and depression and anxiety has been investigated more thoroughly than other mental illnesses like bipolar disorder (BD), obsessive compulsive disorder (OCD), and schizophrenia. PWDs with concomitant depression have lower engagement with self-care activities, like diet, exercise, and taking medications [16]. Similarly, PWDs with anxiety disorders experience increased risk of engaging in poor self-care such as overeating [16, 17]. Thus, these comorbidities show strong evidence of increased morbidity, mortality, increased hospitalizations and health care costs, and a lower quality of life [16]. Considering these findings on the relationship of diabetes and depression and anxiety, further investigation is warranted on the relationship of diabetes with the other mental illnesses. If these relationships are just as additive, costly, and detrimental to healthcare outcomes, further research can guide the deployment of optimized healthcare practice methods, like collaborative care. Improved management and recognition of PWD and BD, OCD, or schizophrenia may improve health outcomes, quality of life, and reduce the economic costs of care as shown with depression and anxiety [18].

Antipsychotics medications are one treatment for schizophrenia; however, many of these drugs have concerning metabolic profiles. These medications can cause an increased risk of diabetes and metabolic syndrome, including weight gain, hypertriglyceridemia, increased levels of insulin, glucose, and low-density lipoprotein cholesterol [19]. Antipsychotic medications are further divided into two categories: first-generation antipsychotics that work by antagonizing dopamine receptors, and second-generation antipsychotics which work through serotonin-dopamine antagonism. Second-generation antipsychotics are also referred to as atypical antipsychotics in clinical settings and research literature [20]. Not only do these medications have different mechanisms of actions, they also have differing adverse effect profiles. Many of the first-generation drugs such as haloperidol come with potential extrapyramidal side effects such as acute dystonia, akathisia, and tardive dyskinesia. The second-generation medications, especially olanzapine, are less likely to promote extrapyramidal side effects, but they come with a significant increase in weight gain and metabolic syndrome [20]. This literature review will examine research about these second-generation antipsychotics and diabetes.

The goal of this review is to analyze and synthesize the literature on diabetes and mental health with a focus on the less studied illnesses, like BD, schizophrenia, and OCD. The aim is to identify and call attention to the bidirectional relationship between mental illness and diabetes to raise awareness and influence change in healthcare practices and precepts. Examining these complex relationships can guide the formation of novel methods of healthcare delivery and collaboration that reduce morbidity and improve outcomes for this population.

## Methods

The intention of the literature review was to explore potential relationships between diabetes and lesser investigated mental health illnesses. This systematic review was conducted by gathering various sources, several of which were retrospective cohort studies that investigated the intersectionality of diabetes and mental illness with SES, epidemiology, and clinical implications. These resources were obtained from March 2023 to February 2024 by online searches through platforms such as the National Library of Medicine, PubMed, Google Scholar, and Articles Plus. The references collected were published from 2003–2023. To isolate information that would be productive to this review we searched for subject headings such as “diabetes”, diabetes mellitus,” “diabetes and mental health illnesses,” and “health outcomes for diabetes and mental illness” as well as searches tailored to specific illnesses addressed in this review. Several of the publications were retrospective cohort studies, others were case-control studies, and cohort studies. No data was quantified for this systematic review so there are no statistical methods that were performed by the authors; however, any statistical methods that were used in the resources gathered have been mentioned in the body of this review and can be further investigated in the original document by the cited author(s).

### Bipolar Disorders

According to the Diagnostic and Statistical Manual (DSM-V), bipolar disorders (BD) are categorized into bipolar I, bipolar II, and cyclothymic disorder. Bipolar I occurs when an individual experiences a manic episode that may have been followed by or preceded by episodes of hypomania or major depression. Bipolar II diagnosis includes experiencing at least one episode of hypomania and at least one episode of major depression without ever having a manic episode. Cyclothymic disorder is a rapid cycling between hypomania and minor depression states. There are also other specified and unspecified categories of BD that do not fit the criteria for bipolar I, bipolar II, or cyclothymic disorder [21]. A large international cross-sectional study found the prevalence of all BDs to be 2.4% worldwide. A 2023 interview study found that almost all BD study participants reported experiencing disruptions to school or work, relationship difficulties, and stigma surrounding their diagnosis [22]. One in every 4 to 5 individuals with BD has previously made a suicide attempt [23].

It is well documented that people with BD have a disproportionately higher lifetime prevalence of both psychiatric and medical comorbidities, like substance use disorders, neurological disorders, and metabolic syndrome [24, 25, 14]. Compared to the general population, people with BD have conspicuously higher prevalence rates of metabolic syndrome [14, 26–29]. A meta-analysis of 16 different cohorts showed that metabolic syndrome is a significant predictor of new-onset diabetes with a relative risk of 3.5–5.2 in those that meet only one metabolic syndrome criteria and a relative risk of 10.88–24.4 for those who meet four or more criteria [30]. The direct link between BD and diabetes is understudied and thus unclear. A systematic review found that the general population’s prevalence of diabetes is 2–3 times greater in those with BD according to three large population cohort studies [29]. This contrasts with two other studies that found no significant correlation between BD and diabetes; however, one study had a small sample size, and the other was only taken from hospitalization data not the general population [25, 31]. A 2022 meta-analysis of 49 separate studies found that the prevalence of T2D in people with BD was 9.6% which is a much higher prevalence than the 6.28% estimation of global general population T2D [32]. Even with the heterogeneity of results, most studies suggest a relationship between BD and metabolic syndrome and some degree of relation between BD and diabetes.

Several studies suggest that the unhealthy lifestyle of people with a mental illness such as BD creates the relationship with metabolic syndrome and diabetes [5, 33, 34]. One potential explanation of this relation is that the stress associated with both diseases can cause cognitive changes that lead to BD [26, 33, 34]. Another explanation may be that people with BD are less likely to regularly get preventative primary care [26, 34, 35]. Others suggest that the medications used to manage BD symptoms play a large role, specifically antipsychotics and antidepressants [26, 28, 33–36].

Research shows that individuals with BD and diabetes are at an increased risk of severe hypoglycemia events or diabetic ketoacidosis (DKA), which can cause micro- and macrovascular complications and even death [35]. The increased risk of acute complications from PWD with BD is theorized to be due to the lack of engagement with diabetes self-management. This is explained by the increased prevalence of barriers in people with BD to effective self-care such as extreme moods, less social support, drug abuse, and low SES [30, 37]. A case report from Taiwan found a correlation between the mood of people diagnosed with BD and T2D and their hemoglobin A1c (HbA1c). During an episode of depression their HbA1c was high, but during a manic episode their diabetes was well managed despite stopping antidiabetic therapy for over 3 months. As they entered another episode of depression, their HbA1c increased once more [38]. Although this case report demonstrates a clear relationship between BD and T2D, it was the only one found in the literature and supports the need for further research. A recent systematic review found a significant relationship between an increased postprandial glucose rate and more symptoms of negative mood in individuals with T2D. Findings included the relationship between glucose variability over multiple days and depressive mood symptoms [39]. With this limited amount of research, it is currently difficult to discern the exact etiology of the bidirectional relationship, but it is clinically relevant to acknowledge its existence, create appropriate guidelines, and improve healthcare provider understanding and communication to prevent adverse health outcomes.

### Schizophrenia

The DSM-V defines schizophrenia as a severe, chronic mental illness that is characterized by disturbances in thought, perception, and behavior. The DSM-V shows a current lifetime prevalence of 0.3–0.7% for schizophrenia with psychotic features first manifesting in individuals between their mid-teen to mid-thirties. To confirm a diagnosis, two or more of the following conditions must be noted for at least one month or longer: delusions, hallucination, disorganized speech, catatonic behavior, and negative symptoms such as apathy and lethargy [40].

Traditional risk factors for T2D are aligned with lifestyle factors such as sedentary activity levels, hypertension, and obesity; however, emerging epidemiological data suggest that individuals with a schizophrenia diagnosis are at a two- to five-fold increased risk of developing T2D [41]. When comparing the traditional risk factor of obesity among those with and without a schizophrenia diagnosis, individuals with schizophrenia are noted to have a two-fold increase in obesity compared with those without a diagnosis [41]. The data collected for this literature review suggests a possible triad of reasoning for these observations. First, the medications available for schizophrenia, particularly second-generation antipsychotics, do not have metabolically favorable profiles [41–43]. Research has suggested that the receptor antagonism activity of serotonin and histamine (H1) are involved as contributing factors to the weight gain experienced by those on antipsychotic drug therapy [34]. Second, individuals with schizophrenia tend to be in a lower socioeconomic bracket and at a greater risk of developing obesity. A 2007 population based longitudinal study showed that when comparing individual and community level SES at the time of the person’s birth, low SES yielded an increased risk of schizophrenia. Socioeconomic status in combination with the poor metabolic profiles of the antipsychotics used as first line treatments in individuals with schizophrenia, the potential correlation to T2D becomes more plausible [44]. Lastly, even with the known potential correlation of T2D with schizophrenia and the increased prevalence of obesity, these individuals have less tests performed for data collection at their primary care visits even though they have more visits than the average population [45–47].

Since their development, antipsychotic drugs have become a staple in the treatment and symptom management of schizophrenia. The safety advantages of second-generation antipsychotics have been questioned and for good reason. These drugs have poor metabolic profiles as demonstrated by their predilection to induce weight gain as well as alter the lipid and glucose profiles of diagnosed individuals [42]. A 2018 meta-analysis investigated the risk of developing diabetes among individuals with schizophrenia that were prescribed antipsychotics. The cohort data showed that the relative risk for developing T2D while on a second-generation antipsychotic (clozapine, olanzapine, risperidone, and quetiapine) was 1.32 with a 95% CI of 1.15–1.51 [43]. Although the review by Smith et al. proposed an increased risk of T2D as high as 30% in those receiving second-generation antipsychotic medications when compared to those receiving a first-generation drug, they did disclose that this number was likely calculated through a biased observation and the true value is likely of a much smaller value [36]. Thus, more research needs to be done towards investigating whether the drug of lifestyle is influencing the development of T2D.

### Obsessive Compulsive Disorder

Obsessive compulsive disorder (OCD) is a condition where individuals can experience obsessions and/or compulsions. The DSM-V defines obsessions as recurrent and persistent thoughts, urges, or even images that are intrusive and unwanted. In response to those obsessions, individuals can develop compulsions which are defined as repetitive behaviors or mental acts that a person feels driven to perform in order to alleviate the intrusive thoughts or urges [40]. The 12-month prevalence of OCD in the United States is currently 1.2% and the international prevalence shows a similar rate of 1.1-1.8% with women being affected at a slightly higher rate than men in adulthood [33]. Individuals with schizophrenia or schizoaffective disorder should also be evaluated for OCD as there is a current prevalence of 12% within this patient population [33].

While there is strong research on the potential links between diabetes and mood disorders, little research is present on possible correlations between diabetes and OCD. A 2022 review by Grassi et al., suggests that insulin signaling, a systemic process triggered by the binding of insulin receptors to aid in metabolism regulation [48], may play a role in the pathophysiology of OCD; however, more research is needed to confirm its true physiology. Research involving deep brain stimulation (DBS) suggests that abnormal dopaminergic transmissions of the striatum may impart impaired insulin sensitivity in individuals with OCD [49]. The limited research available currently suggests that DBS could have the ability to increase insulin sensitivity, but further research should investigate whether current medications such as selective serotonin reuptake inhibitors (SSRIs) could have protective potentials against diabetes related risks in this patient population [49]. The suggestion for further research into SSRIs was proposed based off the results of an Italian cross-sectional study that investigated the prevalence of metabolic syndrome in people with OCD. The observed results suggested a reduced risk of metabolic syndrome when administered in a dose and time dependent manner; however, the authors of the project did not clearly say the reduction was due specifically to the SSRIs because of concerns around the study design and its inability to exclude an indirect effect of SSRIs on metabolic risk [49]. Research on the metabolic impact of SSRIs as well as the symptomatology of OCD in PWD and how it is interconnected with insulin signaling and sensitivity could yield a number of future treatment paths and options for people living with OCD and diabetes [49, 50]. Kontoangelos et al., conducted research investigating the correlations between diabetes, depression, and other OCD symptoms using questionnaires distributed to 131 PWD. Among them, 31 identified as having unregulated blood glucose levels and higher scores for depression and OCD symptomatology. Subsequent follow-up one year later revealed that those who controlled their metabolic profiles showed lower scores for depression and OCD symptoms. The differences in scores between the cohorts provides preliminary data hinting at a potential link between T2D, depression, and OCD symptoms [50].

### Next Steps

Considering the strong emerging evidence found for the efficacy of a collaborative care model in improving the outcomes of depression, anxiety, and comorbid diabetes, it should be considered in the next steps of improving the care of the other mental illnesses [11, 17]. Collaborative care is an evidence-based healthcare model that joins mental health and primary care together with a goal of providing whole-person care to improve outcomes and follow through [11, 16, 17]. This model may prove beneficial for healthcare providers in correctly diagnosing and following up with both diabetes and mental health disorders. Patients with mental illnesses may commonly present with somatic complaints, symptoms that overlap with other disorders, or simply not disclose their psychiatric symptoms due to stigma [3, 5, 51]. Even if a patient is correctly diagnosed, appropriate treatment typically relies on referrals to external psychiatric specialists which poses more challenges. Taking time off work, childcare, transportation, and other costs all pose possible barriers to treatment [17]. Community studies of our current system show that it can take several years for individuals to receive appropriate treatment after onset of their symptoms [11, 51]. Also, the use of psychotropic medication only treats a small portion of an individual’s presentation when considering that mental health issues stem from a combination of biological, psychological, and societal factors [11]. The model of collaborative care appears to remove many of these barriers as it brings primary care providers, psychologists, nurses, and social workers together to ensure patients receive all of the physical, psychological, and social care they need [51]. This is especially important for individuals with comorbid diabetes and a mental health condition since this population has more micro- and macrovascular complications and an increased number of risk factors [44]. The collaborative care model is also particularly advantageous for this medically complicated population because it allows for easy consultation and collaboration of specialists, like endocrinologists and psychiatrists, in clinical decision making [11]. Properly intervening would improve quality of life, improve overall health, and reduce healthcare costs universally [11, 18].

Upwards of 50% of individuals treated for mental illness partially or completely fail to respond to traditional pharmacologic therapy [11]. Emerging studies are finding promise in the use of the antidiabetic drug metformin in treating psychiatric disorders as well as decreasing the metabolic effects of antipsychotic drugs [28, 52]. Metformin is the first line treatment for T2D and works by reducing inslulin sensitivity and decreasing glucose production in the liver [24, 49]. There is robust evidence for the concomitant use of metformin with antipsychotic medications in order to mitigate the weight gain effects of antipsychotics [53, 54]. Antipsychotic-induced weight gain is not only shown to cause medication non-adherence and discontinuation but also can decrease quality of life [55]A 2022 randomized, quadruple-masked, placebo-controlled clinical trial, although small (*n* = 45), investigated using metformin as treatment for patients with insulin resistance who were also diagnosed with BD type I or II. Their results showed that those who became insulin sensitive due to metformin use had a statistically significant decrease in depression and anxiety and they had significant improvement in their daily functioning without an increase in mania [28]. It is unclear whether metformins efficacy in PWD and mental illness is from its weight loss effects, its glycemic control, or both [24]. Regardless, these preliminary studies exhibit the possible role metformin may play in future mental health guidelines and the need for principal comprehensive studies. In addition to metformin, other antidiabetic pharmacotherapies have shown promise in their use for antipsychotic-induced weight gain, most notably glucagon-like peptide-1 receptor agonists [56]However, given the limited amount of research on the subject, it is difficult to provide a comprehensive assessment. Considering the relationship between diabetes and mental illness and the evidence on metformin use, it is reasonable to pursue further investigation into the role of other antidiabetic pharmacotherapies in mental illness.

With such a high prevalence of both diabetes and mental illness compounded with increasing evidence of reciprocal susceptibility and costly consequences, it is imperative to push for more education, research, and guideline changes. It is apparent that there are gaps in research on these lesser studied mental health illnesses and their relationship to metabolic syndrome and diabetes. Research into mental health symptomatology and diabetes management is limited but could be a worthwhile endeavor as it could expand ways to control undesirable symptoms and behaviors. Future investigations are warranted to discern the underlying mechanism behind mental health pharmacotherapies leading to diabetes. It may be possible to combat the adverse metabolic effects of these drugs if their mechanisms are understood. Regardless, more effort is needed to educate healthcare providers on the signs and symptoms of all mental illnesses and the importance of intervention. Further research on the collaborative care model should be done to demonstrate its benefits and efficacy, and providers should consider incorporating a more collaborative, whole-person approach to their patient care. Providers should consider monitoring patients taking psychotropic pharmacotherapies for adverse metabolic effects and patients with diabetes for mental health illnesses.

## Conclusion

The prevalence of type 1 and type 2 diabetes and mental illness continues to rise globally while our current model of fragmented healthcare remains. It is crucial to advocate for greater focus on education, research, and guideline revisions due to the high prevalence of both diabetes and mental illness, along with mounting evidence of their reciprocal susceptibility and costly consequences. Efforts in education should push to equip both healthcare providers and the general public with a broader understanding of the reciprocal nature of these conditions. Additional research must be done to better demonstrate the promise of value that the collaborative care model offers not only to individuals with diabetes and mental health issues, but to anyone with a physical illness and psychological comorbidity. Further research should also be directed towards finding alternative medications with more favorable metabolic profiles and research into the psychiatric benefits metformin may harbor. Concurrently, revisiting existing guidelines is imperative to facilitating more responsive and holistic interventions to improve healthcare outcomes. Through these changes, we can bolster a more resilient healthcare system that can more appropriately serve our communities.

The research collected for this review has yielded many gaps in the care of individuals withdiabetes and mental illness. From the limitation of drug options with poor metabolic profile to the lack of holistic care from healthcare providers, we now have a better understanding of how essential it is for both researcher and healthcare providers to invest time into the correlation of these conditions and how we can work towards improving treatment options, healthcare outcomes, and ultimately quality of life for these individuals. While there are potential limitations with the research referenced in this review, it should serve as a foundation for guiding the future direction of research and understating the complex correlation between diabetes and these lesser addressed mental illnesses. It is our responsibility to ensure the continued development of scientific research and treatment options for all conditions, even those less studied and less prevalent in the general population.

## Data Availability

No datasets were generated or analysed during the current study.

## Abbreviations

ADA: American Diabetes Association
BD: Bipolar Disorder
DKA: Diabetic Ketoacidosis
DSM: Diagnostic and Statistical Manual of Mental Disorders
HbA1c: Hemoglobin A1C
HHS: Hyperglycemic Hyperosmolar State
LA: Lactic Acidosis
OCD: Obsessive Compulsive Disorder
PWD: People With Diabetes
T1D: Type 1 Diabetes
T2D: Type 2 Diabetes
DBS: Deep Brain Stimulation
SSRI: Selective Serotonin Reuptake Inhibitor
